# The determinants of gene order conservation in yeasts

**DOI:** 10.1186/gb-2007-8-11-r233

**Published:** 2007-11-05

**Authors:** Juan F Poyatos, Laurence D Hurst

**Affiliations:** 1 Logic of Genomic Systems Laboratory, Spanish National Biotechnology Centre, Centro Superior de Investigaciones Científicas (CSIC), Darwin 3, Campus de Cantoblanco, Madrid 28049, Spain; 2 Department of Biology and Biochemistry, University of Bath, Bath BA2 7AY, UK

## Abstract

**Background:**

Why do some groups of physically linked genes stay linked over long evolutionary periods? Although several factors are associated with the formation of gene clusters in eukaryotic genomes, the particular contribution of each feature to clustering maintenance remains unclear.

**Results:**

We quantify the strength of the proposed factors in a yeast lineage. First we identify the magnitude of each variable to determine linkage conservation by using several comparator species at different distances to *Saccharomyces cerevisiae*. For adjacent gene pairs, in line with null simulations, intergenic distance acts as the strongest covariate. Which of the other covariates appear important depends on the comparator, although high co-expression is related to synteny conservation commonly, especially in the more distant comparisons, these being expected to reveal strong but relatively rare selection. We also analyze those pairs that are immediate neighbors through all the lineages considered. Current intergene distance is again the best predictor, followed by the local density of essential genes and co-regulation, with co-expression and recombination rate being the weakest predictors. The genome duplication seen in yeast leaves some mark on linkage conservation, as adjacent pairs resolved as single copy in all post-whole genome duplication species are more often found as adjacent in pre-duplication species.

**Conclusion:**

Current intergene distance is consistently the strongest predictor of synteny conservation as expected under a simple null model. Other variables are of lesser importance and their relevance depends both on the species comparison in question and the fate of the duplicates following genome duplication.

## Abstract 2

Current intergene distance is shown to be consistently the strongest predictor of synteny conservation as expected under a simple null model, and other variables are of lesser importance.

## Background

The precise location of genes in eukaryotic genomes was assumed to be largely random not so long ago [1]. This was motivated by the understanding that, unlike in bacteria, there need not be chromosomal domains associated with high rates of gene transcription. Common reports of chromosome inversions with little effect on phenotype confirmed the picture of random placement of genes and a lack of selective constraint on gene order [2].

However, recent studies in diverse eukaryotes challenge this initial intuition [3]. Indeed, in all well studied eukaryotic genomes, genes of similar expression tend to cluster more commonly than expected by chance [3]. For example, in humans both broadly [4,5] and highly [6,7] expressed genes cluster, while in yeast highly co-expressed genes are neighboring more commonly than expected [8]. The same tendency for genes that are physically close to be co-expressed might additionally explain why genes whose proteins are close in either the metabolic [9,10] or protein-protein interaction network [11,12] are in close chromosomal proximity more commonly than expected. More subtle organizations have also been claimed, such as periodicity in gene location [13], but this appears to be caused by data biases [14]. Not all patterns are necessarily associated with co-expression of some variety. Most notably, in yeast, essential genes cluster into domains of low recombination [15]. The clustering of essential genes may be more to do with ensuring precise control over expression (that is, minimal noise), rather than co-expression *per se *[16].

While all these previous analyses helped to clarify some of the factors associated with gene order, they also opened new questions. Most particularly, how important, in absolute and relative terms, are all of these features? If we take a pair of genes adjacent to each other in *Saccharomyces cerevisiae*, we can then ask whether the same two genes are also adjacent or not in a different species. How relevant are the above parameters in explaining which genes are adjacent in both species? In yeast, intergene distance and co-expression have been shown to be two independent determinants of gene order conservation using *Candida albicans *as comparator species [17]. Intergene distance is expected under the simplest neutral null model of gene order evolution. This is because we suppose that a re-arrangement that disrupts a gene will not be tolerated, hence those genes currently with a large intergene distance between them are more likely to be affected by viable gene re-ordering events, all else being equal (note that under this simplest null model it follows that overlapping genes are impossible to break up). Likewise, a pair of genes that currently have a large intergene distance between them are more likely to have had in the past a large intergene distance, even if they were not immediately next to the genes that are currently their neighbors.

Other evidence suggests that this null model alone is not adequate. Notably, essential genes tend to stay together more commonly than expected by chance, although their mean intergenic distance is unexceptional [15,18]. Whether this is owing to selection *per se *or simply a reduced probability of chromosomal re-arrangements in domains of low recombination [12] remains unclear. We can then ask a series of questions. First, if we treat each parameter in isolation, we can ask whether that parameter explains a significant proportion of conservation of gene order. Second, in a fuller model we can ask how relatively important and independent each of the parameters might be. Third, are the results of the above analyses sensitive to which comparator species we employ to compare with *S. cerevisiae*? Fourth, can we predict the characteristics of those gene pairs that through all lineages of yeast have remained physically together? Finally, what will be the effect of the differential gene silencing associated with the whole genome duplication in the yeast lineage?

To address these questions, we computed a group of potential determinants in *S. cerevisiae *and quantified how they determined linkage conservation in a full yeast lineage.

## Results and discussion

### A neutral model of gene order evolution

While in principle a relationship between intergene distance and conservation rates of genes that are immediate neighbors seems reasonable, a problem in demonstrating this derives from the fact that intergene distance data that we can directly obtain from genome sequencing describe the situation after the process of evolution from an ancestor. If we assume that DNA is neither lost nor gained, then a gene pair with a small intergene distance in *S. cerevisiae *may have a small distance either because the pair have always resided together and the intergene distance has not changed or because the pair came together following an inversion and this inversion just happened to bring with it a small intergene spacer. Moreover, two genes may be together in both *S. cerevisiae *and a relatively distant comparator, for example, *C. albicans*, not because they have always been immediate neighbors but because repeated events broke them up but also re-positioned them, bringing them back together. To further investigate the extent to which intergene distance might differ between genes that are immediate neighbors in any two species and those that are immediate neighbors only in one of the two species, we performed a set of neutral simulations.

In these simulations we consider a chromosome with 400 genes. The intergene distance between any gene pair is randomly selected from intergene distances currently observed in *S. cerevisiae *(after removal of overlapping transcripts). We then randomly select a position on the chromosome and accept this position if it is an intergene spacer. We then pick a point that is approximately 5 kb upstream or downstream of the selected chromosome location and accept it as the end point of the inversion if in intergene spacer. This distance approximately matches the mean size of the small inversions seen in yeasts [19]. We then invert the sequence, thereby altering intergene distance between, at the most, two pairs of genes. We then carry on evolving the new chromosome over numerous rounds of inversions. We repeat the simulation for 1,000 inversions 100 times.

**Figure 1:**
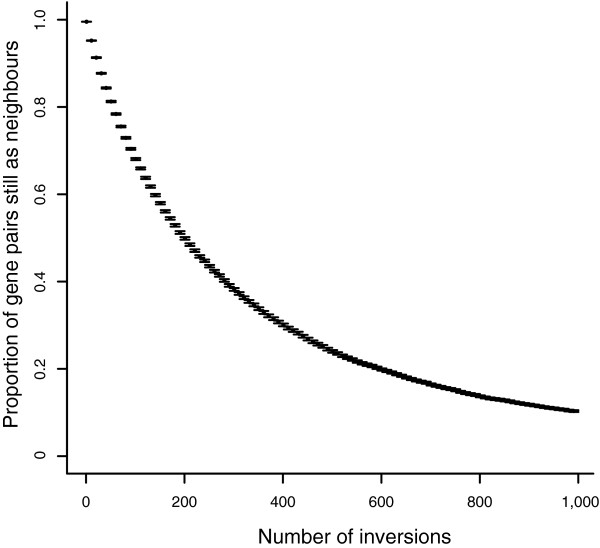
Rate of synteny conservation in a null model of gene order evolution. The relationship between the proportion of gene pairs retained as neighbors and the number of inversions between two taxa.

The first question to ask is what might be the relationship between the number of genes that are still immediate neighbors in the derived chromosome that were ancestrally also immediate neighbors. To examine this we compare the evolved chromosome with the ancestral one and partition gene pairs into those that are in retained synteny (that is, still immediate neighbors) and those that are not. Note that if A and B reside next to each other in the ancestor, then AB or BA ordering is considered to be preserved synteny, regardless of the DNA strand on which the two genes reside. Results are shown in Figure 1. As can be seen, the data describe an exponential decay function of rates of synteny conservation with increasing numbers of inversions. Note too that the asymptote of this function is not zero conserved synteny. This is owing to the fact that by chance in any random chromosome a certain number of gene pairs will be the same as in any other random chromosome.

**Figure 2:**
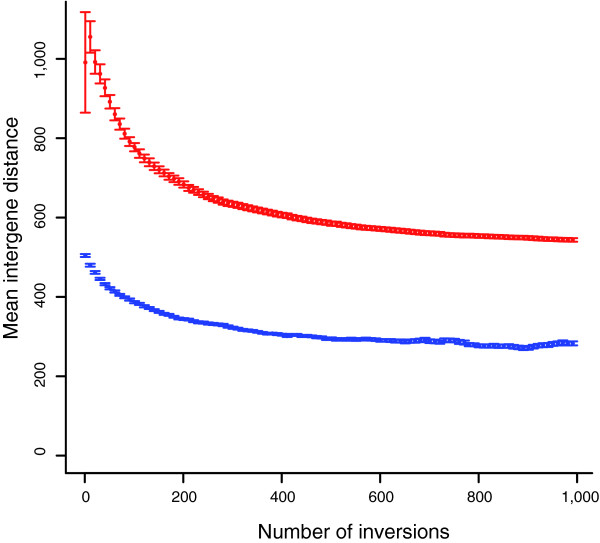
Differences in intergene distance. Intergene distances of gene pairs currently in synteny and in the ancestor (blue) and those that were not ancestral neighbors but currently are neighbors (red) as a function of the number of inversions (error bars are standard error of the mean).

The second question to ask is what, at any given time point following divergence from an ancestor, is the difference in intergene distance between those genes currently in preserved synteny and those that are no longer nearest neighbors. To examine this we compare the current intergene distances between the two groups. For each simulation we consider the mean intergene distance in the two groups and then consider the mean of these means over all simulations. As can be seen (Figure 2), at all divergence times (measured as number of inversions) the group remaining in synteny has a smaller mean intergene distance in the descendent chromosome. At least two reasons underpin this. First, as previously noted, randomly selected positions are most likely in long intergene spacers. A second, less appreciated fact is that when genes separated by a long spacer are involved in inversions, they tend to bring with them abundant intergene spacer sequence. Hence, not only do those genes that are retained in synteny comprise a special subgroup associated with low intergene distance, but those genes not retained in synteny tend not to transfer to the small intergene distance class.

**Figure 3:**
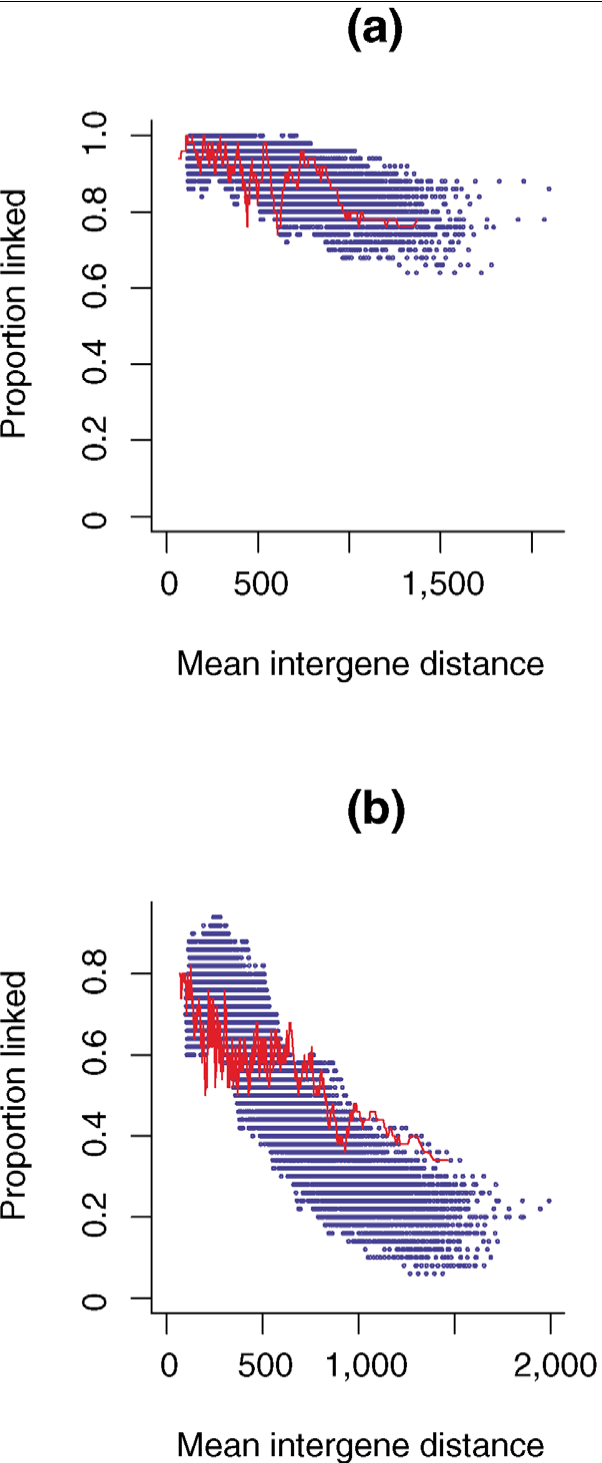
Proportion of gene pairs conserved in a comparator versus intergene distance in *S. cerevisiae*. Profiles of the rate of gene pairs conserved versus their current spacer in *S. cerevisiae *(red) or in simulants (blue) when comparing *S. cerevisiae *with two comparator species for **(a) ***C. glabrata *and **(b) ***A. gossypii*. For the simulations the number of inversions to run was determined by comparing observed synteny conservation rates against inversion number as shown in Figure 1. For our five focal species we also restricted analysis to cases where both of the orthologues of the *S. cerevisiae *gene pair are on the same chromosome in the comparator species, as this fits better the simulant model and permits higher orthology certainty. Each data point in the real and simulant data represents the proportion of gene pairs from 50 showing conserved synteny, after the data was rank ordered by intergene distance. After considering the first 50 we then considered ranks 2-51, 3-52, and so on. In addition, we also considered other comparators, and a much more distant comparator, *C. albicans *(Additional data file 1).

A third question to ask is how the simulations relate to real data. To do this we consider the following for both the real and simulated data. We take all currently observed neighboring gene pairs (that is, those in *S. cerevisiae *or those at the relevant point in the simulations) and rank order them according to their intergene distance. We then consider the top 50 (smallest intergene distance) and ask what proportion are retained in synteny. We then consider ranks 2 to 51 and repeat the calculation and so forth. For each species comparison (*S. cerevisiae *versus other), we consider in the simulations the distributions after the same number of inversions as corresponds to the number of gene pairs overall that are not in preserved synteny in the real data. As can be seen (Figure 3 and Additional data file 1), the correspondence between the simulant data and the observed data is striking. In all cases, for both the real data and the simulation, the genes with the shortest intergene distances show much higher levels of synteny conservation than those with longer intergene distances. This qualitative fit suggests that a simple null model that genes divided by large intergene distances are more likely to be re-ordered or, more precisely, to have been re-ordered, provides, to a first approximation, a good fit. This is yet more remarkable given that we suppose that gene orientation has no impact on the effect of a re-arrangement. This is an unrealistic assumption given that, of all three possible orientations of gene pairs, two convergent genes (→ ←) are unique in having no promoter sequence in the intergene spacer. This might in part explain why in many cases a further regularity appears, namely that gene pairs currently closely linked (that is, with little intergene distance) are not conserved quite as much as in the simulations while, conversely, those with relatively large intergene spacers in *S. cerevisiae *tend to be conserved somewhat more than seen in most simulants, while the overall rate of synteny conservation is the same. Prior evidence, however, also supports the view that the simple null model, even allowing for gene orientation, cannot explain everything. To assess the importance of the other suggested correlates, we consider a set of statistical approaches in which we look for deviations from the null model given by current intergene distance.

### Predictors of gene order conservation

We consider specifically seven factors either previously associated with the formation of clusters or that could predict linkage conservation in *S. cerevisiae*. These predictors are the following: *met*, metabolic relationship [9,10,20]; *cex*, gene co-expression [8]; *igd*, physical proximity (that is, intergenic distance [17]); *let*, density of lethals (that is, local essential gene density) [15]; *rec*, recombination rate [12]; *cre*, gene co-regulation (number of common regulatory motifs between two genes) [21]; and *pro*, distance in the protein-protein interaction network [11,12].

In asking whether the above parameters predict gene order conservation, we could be asking two different questions. First, we could ask whether gene pairs of a particular class, given they are of the same class, are preserved as immediate neighbors more than those not in the same class. Second, we can ask whether, in determining which genes are preserved in linkage, the fact of belonging to the same class can explain much of the variation. The difference in analysis can be easily illustrated. Consider that there were just two genes in the genome that belonged to a given class (perhaps there are just two genes involved in a given cell process, process X). Consider also that these two genes were always preserved in linkage for some reason. At first sight process X looks like a strong predictor, as, given that two genes both belong to class X and are neighbors in one species, we can be sure they are neighbors in another. If we approach the analysis using the first method we would conclude that belonging to class X was important. However, as a variable to explain patterns of conservation or not of gene pairs in general, it explains very little of the conservation of gene order (just one pair) and most conservation of synteny has nothing to do with belonging to class X or not. The second mode of analysis would report that belonging to class X is not an important variable. *A priori *then, we expect the answers to depend on precisely what questions we ask.

We concentrate predominantly on the second mode of analysis. We take two broad approaches. First, for given pairwise comparisons (*S. cerevisiae *versus other species) we ask about statistical models that act to explain the variation between gene pairs as to whether they are syntenic (immediate neighbors) in both species or not. Second, we ask about the properties of gene pairs that are syntenic in all of the species concerned. While the first question allows us to ask whether predictors of conservation of gene order are dependent on the taxa compared and their phylogenetic distance, the second mode permits us to distil the properties that enable gene order conservation in the long term.

To begin, we start by asking whether there is a difference in the predictor variables, each in isolation, between those gene pairs that remain as immediate neighbors and those that do not remain adjacent. To this end, we first computed these values for adjacent gene pairs in the *S. cerevisiae *genome with homologues in a given comparator yeast species. We considered five hemiascomycetes species included in the Yeast Gene Order Browser [22] in which the number of gene rearrangements is not too high. The identification of homologues in a given comparator should take into account the whole genome duplication event that occurred in a shared ancestor of some of the species considered. One has to specifically distinguish between orthologues and paralogues to properly argue about the conservation of a pair as adjacent. To avoid any ambiguous assignment, we used the set of ancestral loci introduced in [23]. Table 1 shows the mean values for these properties for adjacent genes in *S. cerevisiae*, within the previous set, which are found adjacent (-co) or nonadjacent (-nc) in the corresponding comparator. As expected, a significant covariate for gene order conservation in all cases is the intergenic distance, with the physical distance between adjacent pairs in *S. cerevisiae *clearly smaller for those pairs found adjacent in each species comparison. Of the remaining parameters, not all appear as important predictors. While genes adjacent in several species have a stronger co-expression signal and lower recombination rates, by contrast, distance in the metabolic and protein-protein interaction networks did not appear to be a relevant determinant of order conservation. In part this may be a methodological artifact as we assume that all genes not present in the network have an average distance, and most genes are not in the network (a more restricted study, examining only those genes featured in the networks, corroborated their relevance; see Materials and methods). We keep nevertheless both predictors in our study as controls. In summary, this analysis confirms the relevance of several aspects of gene expression control and genetic linkage as predictors of synteny conservation in yeast. This method cannot, however, really quantify the relative importance of each of them, so it is this we describe next.

**Table 1:** Determinants of gene order conservation

	C. glabrata*	S. castelli*	*K. waltii*	*K. lactis*	*A. gossypii*
*Met*-nc	3.831	3.83	3.828	3.828	3.826
*Met*-co	3.83	3.827	3.829	3.829	3.829
*Cex*-nc	0.066	0.068	0.067	0.068	0.067
*Cex*-co	0.077	0.079	0.082	0.086	0.082
*Igd*-nc	528.187	477.038	474.632	471.702	461.193
*Igd*-co	390.175	417.432	370.395	376.93	378.035
*Let*-nc	0.201	0.208	0.206	0.203	0.209
*Let*-co	0.216	0.204	0.226	0.224	0.225
*Rec*-nc	1.07	1.062	1.054	1.056	1.055
*Rec*-co	1.036	1.045	1.029	1.035	1.035
*Cre*-nc	0.207	0.162	0.12	0.129	0.121
*Cre*-co	0.111	0.114	0.113	0.118	0.122
*Pro*-nc	5.371	5.38	5.357	5.35	5.35
*Pro*-co	5.346	5.341	5.34	5.35	5.34
Rate-co	86%	84%	55%	55%	56%
Pairs	1678	1677	1860	1930	1852

Seven features were computed for adjacent gene pairs in *S. cerevisiae *(*met*, metabolic network distance; *cex*, gene co-expression; *igd*, intergenic distance; *let*, density of lethals; *rec*, recombination rate; *cre*, gene co-regulation; *pro*, protein-protein interaction network distance; see Materials and methods). The table shows the mean value of each of these properties for adjacent gene pairs that remained (-co) or did not remain (-nc) as adjacent in the corresponding comparator yeasts. Species ordered according to phylogenetic distance to *S. cerevisiae*, the closest being *C. glabrata*. Note that there does not exist yet a consensus phylogeny, for example [22,18]. The last two rows list the proportion of adjacent *S. cerevisiae *pairs retained as adjacent in the corresponding comparator and total number of homologues pairs (both orthologues might not be in the same chromosome). A smaller number was obtained (9% and 1,850 homologue gene pairs, respectively) when using *C. albicans *as comparator species [17]. *Yeasts that diverged after the whole genome duplication event.

### Quantifying predictor relevance in single species

We use multivariate analysis to disentangle the contribution of each of the previous factors to gene order conservation. The general idea is to describe the relationship between a dependent variable, the response, and a group of independent variables, the predictors, by means of a multiple regression model. In our case the response variable takes two discrete values; an adjacent gene pair in *S. cerevisiae *could be found as adjacent or nonadjacent in a given comparator species, so we apply logistic regression [24]. We consider several complementary strategies to estimate the relevance of each linkage predictor. In addition, since the correlation between some of the determinants could also be relevant, for example, essential clusters having low recombination rates [15], a phenomenon termed multicollinearity in regression modeling, we compute the correlation matrix of the parameter estimates in the logistic equation to quantify this effect (Materials and methods). The final outcome of all these combined studies is the simplest logistic model capable of predicting the observed conservation patterns.

We first apply these methods to the closest comparator species, *Candida glabrata*, a post whole genome duplication (WGD) species (Table 2). In the univariate regression studies, the residual deviance of a logistic model with a single covariate is shown. We find a deviance value smaller than that of the null case (dev.*null *= 853.06) for some of the variables, notably co-regulation (dev.*cre *= 845.2). This indicates the possible relevance of this factor as a predictor. The strength of each factor is further supported by the order of appearance of the corresponding variable in a forward stepwise regression model. This method includes as part of the descriptive model only those terms that increase the goodness of fit (according to the Akaike's criterion). In the multiple regression analyses, we present estimates of the regression coefficients related to each predictor with their corresponding standard errors (*z*-values). The last two subcolumns in this study give the deviance table, differences between models as variables are added to the model in turn, and the probabilities associated with an approximated *χ*^2 ^test (deviance differences have approximately a *χ*^2 ^null distribution with degrees of freedom equal to the difference between the numbers of parameters in the two models [24]). After this combined study, we introduce a reduced model in which we retain only co-regulation and intergenic distance as significant determinants. Indeed, if we compare the full model, including all variables, with the reduced one, we can hardly notice the difference in coefficient estimates or deviance (data not shown). This is striking as it suggests that, for this particular analysis, most of the proposed co-variates are too weak to register as explanatory variables.

**Table 2:** *S. cerevisiae *versus *C. glabrata *logistic regression analyses

			Multiple regression			
			
	Simple regression	Stepwise regression	Estimate	z-value	Residual deviation	*P*(>|χ|)
Null	853.06	(0) 855.07	2.535	25.310	853.07	-
*Met*	852.93	(-)	-0.052	-0.692	852.94	-
*Cex*	852.35	(-)	0.05	0.526	852.22	-
*Igd*	833.09	(1) 837.09	-0.312	-4.172	832.48	<0.0001
*Let*	853.02	(-)	0.044	0.453	832.33	-
*Rec*	850.4	(-)	-0.084	-0.935	830.95	-
*Cre*	845.2	(2) 835.1	-0.168	-2.08	827.16	<0.05
*Pro*	852.7	(-)	-0.092	-0.995	826.22	-

The first column lists the seven predictors contributing to the generalized models and the corresponding null model. The second column shows residual deviance (equivalent to the residual sum of squares in ordinary regression analyses) of a model with a single determinant. The third column describes a stepwise forward regression according to the Akaike criterion with insertion order in parenthesis. The last four columns list the results of a multiple regression model (estimates and *z*-values) and the corresponding Anova with terms added sequentially from *met *to *pro *(residual and *χ*^2 ^test).

The relationship between the probability that an adjacent pair in *S. cerevisiae *was adjacent also in *C. glabrata*, *Pr*, and their intergenic distance and co-regulation is:

*logit Pr *= 3.028 - 0.001 *igd *- 0.526 *cre*

where the logit transformation is given by logit $Pr=\frac{Pr}{1-Pr}$, and *igd*, *cre *denotes intergenic distance (in units of base-pairs) and co-regulation score (with a maximum value of 1), respectively. The model indicates that the probability to be adjacent in both species decreases with intergenic distance and co-regulation, the latter being the weaker of the two determinants. The relevance of each variable is easily determined by comparing the coefficients in Table 2, where variables were scaled in standard deviations (standardized data). A higher absolute value of an estimate in these units, and its order of appearance in the stepwise regression, reflects this relevance. Moreover, the previous model gives us the effect of change in one determinant when controlling the other. Thus, the effect of an increase in intergene distance (in units of base-pairs) for a fixed co-regulation score is exp(-0.001) = 0.999, while the maximal effect of increase in co-regulation, controlling for spacer, is exp(-0.526) = 0.591 with *cre *= 1. Are these effects independent? Analyzing the correlation between estimates, we find some dependence between both predictors (correlation of *cre*-*igd *coefficients: -0.19). We could in turn add an additional term in the model to account for this interaction. However, the decrease in deviance achieved by this more complex model is small, so we can still consider the two-component model as a valid description. Overall, this corroborates that an increase in intergene distance diminishes the probability that genes are adjacent in both species. This also suggests that non-adjacently conserved pairs exhibit stronger co-regulation, which is, at first sight, a counter-intuitive result. Analyzing this behavior in detail (data not shown), we find that high co-regulation scores are associated with a low density of regulatory motifs, that is, regulation by a small number of common transcriptional factors. It is probably this low density of regulatory sites that ensures that any re-arrangement is less likely to be opposed by purifying selection.

### Predictors of linkage conservation in a yeast lineage

How would the previous model change with the comparator species? To analyze this question, we consider five comparator species: *C. glabrata *(discussed above), *Saccharomyces castelli*, *Kluyveromyces waltii*, *Kluyveromyces lactis*, and *Ashbya gossypii*. We apply the same methodology as in the previous section (Additional data file 1) to obtain the corresponding reduced logistic models. These models include only those terms which significantly contributed to explain the conservation pattern.

Table 3 shows the models associated with the different comparators. We see that recombination rate and, especially, co-expression emerge as new determinants for pre-WGD species. We examined how the probability to remain adjacent changes with the comparator for the situation of an adjacent pair with null spacer and averaged features, that is, zero mean co-expression, no co-regulation and averaged recombination rate (*rec *= 1). These probabilities are (see Table 3):

**Table 3:** Logistic models of gene order conservation for different comparator species

Species	Model
*C. glabrata*	*logit Pr *= 3.028 - 0.001 *igd *- 0.526 *cre*
*S. castelli*	*logit Pr *= 2.246 - 0.0005 *igd*
*K. waltii*	*logit Pr *= 1.159 + 0.741 *cex *- 0.001 *igd *- 0.422 *rec*
*K. lactis*	*logit Pr *= 0.62 + 1.047 *cex *- 0.001 *igd*
*A. gossypii*	*logit Pr *= 0.753 + 0.849 *cex *- 0.001 *igd*

We applied a combination of methods (see main text) to obtain the simplest logistic model capable to describe the observed conservation. Here *Pr *is the probability that an adjacent pair in *S. cerevisiae *is found adjacent in the corresponding comparator with logit $Pr=\frac{Pr}{1-Pr}$. *Cex*, co-expression; *cre*, co-regulation score; *igd*, intergenic distance; *rec*, recombination rate.

$$Pr(C.\;glabrata)=\frac{1}{1+exp(-3.028)}=0.95$$

$$Pr(S.\;castelli)=\frac{1}{1+exp(-2.246)}=0.91$$

$$Pr(K.\;waltii)=\frac{1}{1+exp(-1.159-0.422)}=0.83$$

$$Pr(K.\;lactis)=\frac{1}{1+exp(-0.62)}=0.65$$

$$Pr(A.\;gossypii)=\frac{1}{1+exp(-0.753)}=0.68$$

Thus, an 'averaged' adjacent pair in *S. cerevisiae *with null intergenic distance is less likely to be found as adjacent in a given comparator as phylogenetic distance increases. While this is, naturally, trivial, it goes some way to validating the method. More interesting is to see how this behavior changes when these pair types have non-zero intergenic distances? For a characteristic spacer of 500 bp, the probabilities to remain adjacent are (0.93, 0.85, 0.75, 0.53, 0.56), which correspond to a percentage of decrease with respect to previous values of ~(2.1%, 6.6%, 9.6%, 18.5%, 17.6%). Gene pairs with a large intergene distance should then be disproportionately more likely to be conserved as adjacent the closer the comparator species is to *S. cerevisiae*. Put differently, as the time to common ancestor increases, as intergene distance increases, so the probability that the genes are not in synteny in the comparator goes up at an accelerating rate.

Co-expression and intergenic distance act as the two main determinants of order conservation in pre-WGD species (clustering of essential genes near the adjacent pairs appears as a third determinant in *K. waltii*). These variables appear to be independent since the correlation of their corresponding estimates is low in all proposed models (<0.08 in all three pre-WGD comparators). To analyze the role of co-expression in more detail, we discretize the co-expression values so that a unit increase in the model corresponds to an increase of 0.1 in the correlation (estimates did not change very much with respect to those in Table 2). What is the effect of an increase in co-expression? This is given by the exponential of the corresponding coefficient in the logistic model. For *K. waltii*, this reads as exp(0.78) = 2.18, which indicates that, controlling for intergene distance and recombination rate, each increase in the correlation of 0.1 increases the odds that a pair remains as adjacent by 2.18 (slightly higher values applied to *K. lactis *and *A. gossypii*).

### Properties of gene pairs preserved throughout yeast evolution

**Figure 4:**
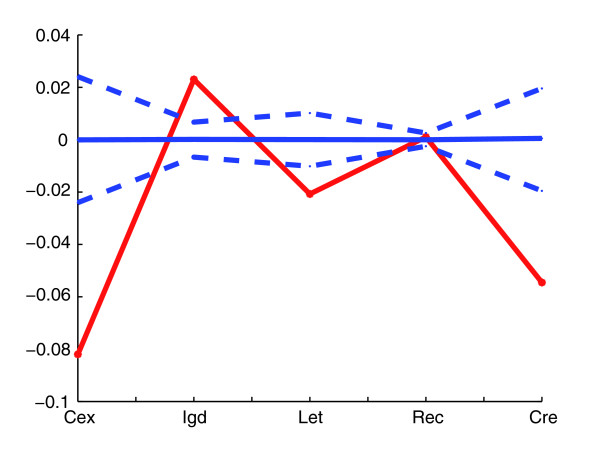
Determinants of close non-adjacently conserved pairs versus distant adjacently conserved pairs. The difference between the ratio of determinant values of non-adjacently conserved genes in a close species to *S. cerevisiae *(*C. glabrata*) and those adjacently conserved in a distant species (*A. gossypii*) is plotted in red for each predictor (line between points to help visualization). This ratio is defined as the quotient between the corresponding values of the close (distant) pairs and those of the adjacently conserved pairs in the close species, that is, *C. glabrata*. We also plotted the null behavior obtained by random sampling of the combined group, close and distant, preserving group size, 10,000 times (mean, continuous blue line, ±2 standard deviations, dashed blue lines). Behavior was qualitatively robust for the *cex*, *igd*, and *let *predictors when using *S. castelli *and *K. lactis *as close/distant comparator (Additional data file 1).

Rather than asking whether given variables explain conservation of order in a given pairwise comparison, for which, as we have seen, the answer heavily depends on the species chosen, we can also ask which parameters are relevant to gene order conservation by considering only those gene pairs that are immediate neighbors through all the species we have examined (509 pairs). Naturally, the answers will again be somewhat subject to precisely which species we consider (indeed, consider a comparator as distant as *Schizosaccharomyces pombe *and no gene pairs are conserved among all species). Nonetheless, this method allows us to distil the factors that are consistently important across a long evolutionary time period. The mean values of the determinants associated with this set were compared to those obtained by randomly selecting a group of genes of the same size (this was obtained by sampling from the full set of *S. cerevisiae *adjacent genes with homologues, adjacent or not, in at least one comparator species, 10,000 times). Adjacent pairs that have been retained as such in the whole lineage exhibited higher co-expression (0.0843 versus a random value of 0.0767, *P *< 0.05), smaller intergene distance (344.52 bp versus a random spacer of 422.44, *P *< 0.0001), higher density of essential genes nearby (0.25 versus a random density of 0.21, *P *= 0.0003), smaller recombination rate (1.029 versus a random rate of 1.043, *P *< 0.05) and lower co-regulation (0.093 versus a random score of 0.125, *P *< 0.01). These results thus support the view that the importance of predictor variables is in the order: intergene distance > local density of essential genes > co-regulation > co-expression and recombination rate. The lineage effect can be alternatively quantified by comparing the nominal values of the determinants of those genes remaining non-adjacent in the least distant species to *S. cerevisiae*, *C. glabrata*, with those of the genes retained as adjacent in the most distant species, *A. gossypii*. This corroborates the relevance of co-expression, density of flanking essential genes and intergene distance in the conservation of pairs in the lineage (Figure 4).

Given that clusters of essential genes also have low recombination rates, we can, in addition, ask whether the retention of synteny of those gene pairs in the middle of essential gene clusters is due to the low recombination rate. In wheat, for example, it is observed that chromosomal domains associated with low recombination rates also have low re-arrangement rates [25], potentially consistent with a model in which recombination is associated with the generation of re-arrangements. By contrast, recent simulations suggest that selection to preserve essential genes in chromosomal domains of low gene expression noise (open chromatin) will result in low rates of disruption of gene order in essential gene clusters, independent of any effect of recombination [16]. To ask whether essential gene clusters are conserved owing to their low recombination rates, we considered two subclasses: those gene pairs with very few essential genes in their vicinity (the 'low' group: *N *= 385) and those with many (the 'high' group: *N *= 34). Next, within each group we ask about the recombination rate of those pairs conserved in synteny across all lineages and those not so. If recombination is an independent predictor, then in both the high and low groups the recombination rate of those in conserved synteny should be lower. For the 83 pairs in the low group conserved as a pair, the recombination rate is slightly lower than that of the low group as a whole (1.04 versus 1.06), but not significantly so (*P *= 0.5). In the high class, 10 of the 34 are retained in synteny. These have, if anything, a slightly higher recombination rate than the average for the high group (0.98 versus 0.95), but again, not significantly so (*P *= 0.47). So, in sum, while clusters of essential genes have low recombination rates, the recombination rate does not in and of itself explain the conservation of synteny. This is not to say that what is reported in wheat is wrong nor, indeed, more generally that recombination does not induce re-arrangements. The most important problem in this analysis is that the recombination rate measurements come from current laboratory yeasts while the synteny conservation data spans events over hundreds of millions of years. Problematically, recombination rates are thought to evolve quite fast. To better resolve this issue it might be better to compare telomeric (high recombination rate) and centromeric (low recombination rates) domains, rather than asking about conservation of gene pairs in isolation.

### Predictors of linkage conservation and reciprocal gene loss

How would the conservation of synteny of a given pair of neighboring genes be influenced by the processes associated with the WGD event? We focus our attention on two possible effects. First, linkage conservation might be influenced by the fate of the pre-WGD adjacent pairs after the WGD event. We could compare two opposite situations. Either both adjacent pairs have lost the same (orthologue) copy of the corresponding gene, or both remained duplicated in all three post-WGD species. These are actually the most common fates of ancestral loci in yeasts [23]. According to this, one could imagine, for instance, that since a duplicate gene might contribute to perform part of the function originally associated with a single gene (sub-functionalization model), adjacent genes with duplicates could experience less pressure to remain linked, as part of the function is implemented by the duplicate. We would predict then that adjacent pairs resolved as single copy in all post-WGD species would more often be found as adjacent in pre-WGD species. This is indeed what we obtain. Single copy adjacent genes were more likely conserved as adjacent in all pre-WGD species: *K. waltii *(*χ*^2 ^= 5.83, *P *< 0.02, d.f. = 1); *K. lactis *(*χ*^2 ^= 5.77, *P *< 0.02, d.f. = 1); and *A. gossypii *(*χ*^2 ^= 5.41, *P *= 0.02, d.f. = 1).

Alternatively, as deletion of one duplicate is the most common process after the WGD, linkage conservation could be influenced by how this deletion is resolved in the different post-WGD lineages. Divergent classes are those in which some of the genes lost are paralogues in the three post-WGD species, while convergent classes imply that all lost genes are orthologues. This latter class implies a less random choice of gene loss. We find that adjacent pairs both belonging to the convergent class are more conserved than expected in four out of five species: *C. glabrata*, *χ*^2 ^= 4.18, *P *= 0.04; *K. waltii*, *χ*^2 ^= 6.64, *P *= 0.01; *K. lactis*, *χ*^2 ^= 9.56, *P *< 0.01; *A. gossypii*, *χ*^2 ^= 6.81, *P *< 0.01; d.f. = 1 in all cases.

## Conclusion

In asking about what factors determine gene order conservation, despite the dependence of the answer on the question, one regularity appears. This is the finding that gene pairs currently with a short intergene spacer are less likely to have been re-arranged. This fits with data from microsporidians in which gene overlap is common and gene order rearrangements are rare [26]. The null model, assuming nothing more than an intolerance to inversions that cut within genes, provides a strikingly good fit for such a simple model. The model was made deliberately simple by not assuming that gene orientation would make a difference and takes no account of the density of functional sites between genes. As noted above, these are unrealistic assumptions. This indeed may explain in part the conservation of gene pairs that are co-expressed, as inversions could, for example, break bidirectional promoters between genes in divergent orientation.

Beyond the role of the intergene spacer, further answers are dependent on just how one asks the question. We can, for example, ask whether gene pairs in a given class tend to be more conserved than gene pairs not in the specified class. For example, gene pairs that specify proteins close in either the metabolic or protein interaction network do tend to be more commonly conserved as neighbors than gene pairs that also specify proteins that feature in the relevant network but are not close in the network. By contrast, if we ask whether network proximity is generally an important predictor of synteny conservation, the answer is no, largely because most proteins do not explicitly feature within the network. Second, when asking about predictors of linkage conservation, the answer depends on which species one is comparing. Close comparators highlight co-regulation, whilst more distant comparators suggest co-expression and maybe the recombination rate (as measured in *S. cerevisiae*) as important predictors. Analysis of the properties of the gene pairs preserved as a pair in all species points to the density of flanking essential genes as an important predictor, suggesting that essential gene clusters tend to be frozen, as previously noted [15,18].

That the results are dependent on the species under comparison perhaps reflects a difference in the strength of selection to preserve a class of gene pairs and the commonality of such pairs. Consider, for example, the possibility that the top 2% of co-expressed gene pairs are under very strong selection to remain linked. Would this be transparent in comparisons between closely related species? The answer is probably not. In our close comparators, approximately 90% of gene pairs remain as immediate neighbors. If just the 2% most highly co-expressed genes resist re-arrangement, there may not even have been a single re-arrangement that might have occurred between linked highly co-expressed genes that was rejected by selection. Hence there would be no signal of co-expression as an important factor in linkage conservation. As the distance between comparators increases, however, the resilience of the 2% will start to appear as an ever stronger signal, assuming the co-expression to be both ancestral and under selection (in different ecologies different co-expression profiles might be under selection). In sum, strong but relatively rare selection will be discernable only in distant comparators. Put differently, the more distant comparisons and the analysis of those pairs always conserved hones in on the special subclass of genes for which selection acts to preserve the gene order.

Perhaps then relatively little is to be learnt from relatively close comparators as so few re-arrangements will have been sampled. In this context, however, there exists one apparent oddity. In the close species comparisons intergene distance and co-regulation appear as important predictors. However, against expectations, gene pairs with a high level of co-regulation, that is, that share much of the same transcription factor-based regulation, are more, not less, likely to be broken up. When analyzed in detail, however, we find that this strong signal is associated with a low density of regulatory motifs: very high co-regulation scores are disproportionately associated with gene pairs with only one (the same) transcriptional motif, hence a low motif density. It is this low motif density that most likely contributes to the lack of conservation of the gene pairs in the short term.

Even if we assume that longer distance phylogenetic comparisons are best, the yeast analysis suggests that phylogenetic distance alone is not the sole arbiter. Rather than the comparator distance, the duplication event experienced in the lineage seems also to be influencing the fate of adjacent pairs. The potential relaxation of the functional constraints associated with the pair members, because of either being duplicated or being divergently conserved, is reflected in a smaller tendency to remain as immediate neighbors.

The results presented here no doubt do not reflect the full complexity of gene order evolution. For example, while we expect that the absolute rate of gene order evolution should scale monotonically with the amount of intergene spacer, this model fails to make any sense of the much higher re-arrangement rates seen in rodents than in primates [27], although the low rate seen in chicken is consistent, the chicken genome being relatively compact. We can also ask whether the other forces we have identified might have any general applicability? Prior reports have found that clusters of housekeeping genes in mammalian genomes tend to have preserved synteny [28] and that essential gene clusters in mice are also conserved [29]. In these instances it will be informative to ask about the relationship between the two parameters (there is a broad overlap between essential genes and housekeeping genes in mammals) and how intergene distance and recombination rate might interrelate. More generally, when more whole genome dispensability data are available it will be interesting to see if the preservation of essential clusters is a common phenomenon and, in turn, ask about the underlying rationale.

## Materials and methods

### Comparator species and genome data

We used data from the Yeast Gene Order Browser [22]. This collection includes seven hemiascomycetes species, four of them having diverged before the whole genome duplication event occurred in the lineage. We considered only six for our study, three pre-WGD, that is, *A. gossypii*, *K. lactis *and *K. waltii*, and three post-WGD, that is, *C. glabrata*, *S. castelli *and *S. cerevisiae*. To compute unambiguosly whether a given syntenic pair in *S. cerevisiae *is conserved as syntenic in a comparator species we analyzed only pairs from a subset of genes termed ancestral loci. Each member of this set correspond to a locus in a pre-WGD species, or the corresponding duplicated pair of loci in the post-WGD species and has been defined by homology and genome context information [23].

### Metabolic network

We examined the metabolic relationship of a gene pair by means of a metabolic network of *S. cerevisiae *recently reconstructed using genomic, biochemical and physiological information [30]. More specifically, we considered this network as a graph whose nodes and edges are the metabolic genes and the metabolic reactions, respectively [20], and quantified the metabolic relationship of a pair by its shortest distance in the network (graph has 851 nodes, and 294 of them are ancestral loci). We computed how many syntenic pairs belonging to this network (up to three intervening genes between them in *S. cerevisiae*) are conserved as syntenic (non-syntenic) in *C. glabrata*. We found 29 genes conserved in *C. glabrata*, 4 of which are non-syntenic. The mean graph shortest distance of those conserved (not conserved) as syntenic is $\bar{d}$ = 3.58 ($\bar{d}$ = 5). This hints at metabolic network distance as a plausible predictor of linkage conservation, that is, the closer in the graph the more likely to be conserved as a linked pair corroborating previous studies [9,20]. For the extended study with all syntenic genes included, we assigned a null distance value to those adjacent pairs without metabolic information. We set this characteristic value to the network mean value ($\bar{d}$ = 3.83).

### Co-expression and intergenic distance

To quantify gene co-expression, 40 different sets of genome-wide transcription time series from ExpressDB were used as compiled in [31]. In our analyses co-expression for a given gene pair denotes then the mean of the 40 correlation coefficients of mRNA expression, corresponding to 40 different experiments, for a given gene pair. All sequence information was obtained from the *Saccharomyces *Genome Database [32].

### Density of lethals

We used a list of essential genes included in the *Saccharomyces *Genome Database [32], which contains information on a large-scale knockout study [33]. We introduced a score quantifying the number of essential genes around a syntenic pair. For each pair, the density of lethals reads as the mean number of essential genes located at -3,-2,-1,pair1,pair2,1,2,3 gene coordinates, that is, up to 4 genes in either the 5' to 3' or the 3' to 5' direction around each member of the pair.

### Recombination rate

We considered the recombination data set obtained in [34], an estimate of recombination rate, by using double strand break analysis. To each gene we assigned a recombination rate. For a syntenic pair we took the mean of each gene rate.

### Co-regulation

We used a dataset of regulatory motifs determined in [35]. A *P *value threshold of 0.001 was considered to select the transcriptional factor binding sites. The co-regulation of a pair is denoted by the number of common regulatory motifs between two genes [31]:

$$O=\frac{\left|m_{1}\cap m_{2}\right|}{max(|m_{1}|,|m_{2}|)}.$$

Here |...| denotes the size of the set, ∩ the intersection and m_*i *_the number of regulatory motifs for gene *i *in the pair.

### Protein-protein network

We used the high confidence data set of multi-validated protein interactions in *S. cerevisiae *[36] and quantified the protein-protein relationship of a pair by its shortest distance in the network as before. Only 168 adjacent genes are included in this graph (with 1088 nodes). We found 155 genes conserved in *S. castelli*, 18 of which remained adjacent. The mean graph shortest distance of those conserved (not conserved) as adjacent is $\bar{d}$ = 5.12 ($\bar{d}$ = 5.28). This hints at protein-protein network distance as a plausible predictor of linkage conservation, that is, the closer in the graph the more likely to be conserved as a linked pair. For the extended study with all adjacent genes included, we assigned a characteristic distance value to those adjacent pairs without protein-protein interaction information. We set this characteristic value to the network mean value ($\bar{d}$ = 5.35).

### Logistic regression

Logistic regression is a class of multivariate analyses usually applied to describe the dependencies of binary responses with respect to a set of variables [24]. While multiple regression usually quantifies the influence of several factors on continuous dependent variables, logistic regression extends these techniques to the study of qualitative features. In our case, we interpreted an adjacent pair in *S. cerevisiae *retained or not as syntenic in a given comparator as such a binary, or discrete, response to model. To estimate the relevance of each linkage predictor in a robust way, given that genomic data are known to be noisy and analysis of them might lead to very significant but totally misleading results [37], we considered several complementary strategies: simple (logistic) regression of each of the variables; forward stepwise regression according to the Akaike criterion (a measure of goodness of fit) [24], in this case, predictors are included in the model only if they increase the goodness of fit; multiple regression - here all proposed variables are considered; and principal component multiple regression (Additional data file 1). We scaled each of the covariates to zero mean and unit variance (standardized data) before carrying out the studies unless indicated. Correlation between some of the determinants could be relevant, for example, essential clusters having low recombination rates. To quantify the correlation between independent variables, one can compute the correlation matrix of the parameter estimates in the logistic equation. Large values of these correlations indicate that multicollinearity may be complicating the modeling process. Alternatively, if correlations between the estimates are fairly small, it is expected that removing one variable from the model does not change the coefficients and *P *values for other variables much.

## Abbreviations

*cex*, gene co-expression; *cre*, gene co-regulation; *igd*, intergenic distance; *let*, density of lethals; *met*, metabolic relationship; *pro*, distance in the protein-protein interaction network; *rec*, recombination rate; WGD, whole genome duplication.

## Authors' contributions

JFP and LDH conceived and designed the study, analyzed the data, and wrote the paper. LDH implemented the null models, JFP implemented the statistical models.

## Additional data files

The following additional data are available with the online version of this paper. Additional data file 1 is an extended discussion of the regression and null models corresponding to several comparator species.

## Supplementary Material

Additional data file 1 Includes the extended discussion, a complementary figure related to the determinants of close non-adjacently conserved pairs versus distant adjacently conserved pairs, and a table summarizing the results of the principal component regression analysis. Click here for file
