# Supplementary material for: The determinants of gene order conservation in yeasts
Source: Genome Biol. 2007 Nov 5;8(11):R233. doi: 10.1186/gb-2007-8-11-r233 (PMC2258174; doi:10.1186/gb-2007-8-11-r233)
Supplement: Additional data file 1 — Includes the extended discussion, a complementary figure related to the determinants of close non-adjacently conserved pairs versus distant adjacently conserved pairs, and a table summarizing the results of the principal component regression analysis. [file gb-2007-8-11-r233-S1.pdf]

Supplementary Information

The determinants of gene order conservation in yeasts

Juan F. Poyatos and Laurence D. Hurst

Here we present additional results for the regression (Tables S1–S4) and null (Figures S1–S4) models corresponding to several comparators species. We also include a complementary figure (Fig. S5) related to the discussion about the determinants of close non–adjacently conserved pairs vs. distant adjacently conserved pairs (Fig.4, main text). Finally, we show a table summarizing the results of the principal component regression analysis (Table S5).

	Simple Regression	***Stepwise Regression***	***Estimate***	***Multiple regression*** z***–value***	***Res. Dev.***	***P(***$> \|\chi\|$***)***
null	1131.19	(0) 1133.19	–	–	1131.19	–
met	1130.98	(-)	-0.057	-0.587	1130.98	–
cex	1130.20	(-)	0.06	0.762	1129.99	–
igd	1126.37	(1) 1130.37	-0.136	-1.936	1125.38	$<$0.05
let	1130.86	(-)	-0.04	-0.509	1125.08	–
rec	1130.21	(-)	-0.060	-0.776	1124.26	–
cre	1128.3	(-)	-0.098	-1.328	1122.74	–
pro	1129.88	(-)	-0.098	-1.293	1121.14	–

Table S1: *S. cerevisiae* **vs.** *S. castelli* **logistic regression analyses**. The first column lists the seven predictors contributing to the generalized models and the corresponding null. The second column shows residual deviance (equivalent to the residual sum of squares in ordinary regression analyses) of a model with a single determinant. The third column describes a stepwise forward regression according to the Akaike criterion with insertion order in parenthesis. Finally, the last (multi)column is the result of a multiple regression model (sub-columns estimates and z-values) and the corresponding Anova with terms added sequentially from met to pro (subcolumns residual and χ test).

	Simple Regression	*Stepwise Regression*	*Estimate*	*Multiple regression* *z–value*	*Res. Dev.*	*P(> $\|\chi\|$)*
null	2416.05	(0) 2418.05	–	–	2416.05	–
met	2415.95	(-)	0.01	0.218	2415.95	–
cex	2410.23	(2) 2366.34	0.085	1.718	2410.11	$<$0.05
igd	2363.9	(1) 2367.9	-0.374	-6.616	2360.29	$<$0.0001
let	2414.98	(-)	0.037	0.749	2359.37	–
rec	2409.87	(3) 2365.05	-0.09	-1.826	2356.38	$<$0.05
cre	2415.84	(-)	0.065	1.280	2354.59	–
pro	2415.42	(-)	-0.052	-1.060	2353.46	–

Table S2: *S. cerevisiae* **vs.** *K. waltii* **logistic regression analyses**. Columns description as Table S1.

	Simple Regression	*Stepwise Regression*	*Estimate*	*Multiple regression* *z–value*	*Res. Dev.*	*P(> $\|\chi\|$)*
null	2386.82	(0) 2388.82	–	–	2386.82	–
met	2386.78	(-)	-0.003	0.053	2386.78	–
cex	2377.56	(2) 2346.15	0.13	2.604	2377.53	$<$0.001
igd	2347.39	(1) 2351.39	-0.312	-5.627	2340.12	$<$0.0001
let	2383.76	(3) 2345.53	0.073	1.457	2337.50	–
rec	2382.42	(-)	-0.065	-1.307	2335.90	–
cre	2386.4	(-)	0.039	0.758	2335.30	–
pro	2386.82	(-)	-0.01	-0.198	2335.26	–

Table S3: *S. cerevisiae* **vs.** *K. lactis* **logistic regression analyses**. Columns description as Table S1.

	Simple Regression	*Stepwise Regression*	*Estimate*	*Multiple regression* z*–value*	*Res. Dev.*	*P(*$>|\chi|$*)*
null	2275.16	(0) 2277.16	–	–	2275.16	–
met	2274.73	(-)	0.022	0.441	2274.73	–
cex	2269.27	(2) 2246.09	0.1	1.943	2268.84	<0.05
igd	2244.61	(1) 2248.6	-0.29	-5.198	2239.78	<0.0001
let	2272.47	(3) 2245.77	0.07	1.358	2237.46	–
rec	2271.28	(-)	-0.064	-1.275	2236.03	–
cre	2275.15	(-)	0.072	1.365	2234.06	–
pro	2274.89	(-)	-0.037	-0.733	2233.52	–

Table S4: *S. cerevisiae* **vs.** *A. gossypii* **logistic regression analyses.** Columns description as Table S1.

	C. gla	*S. cas*	*K. wal*	*K.lac*	*A. gos*
met	0.067	-0.057	0.025	-0.027	0.025
cex	0.086	0.079	0.102	0.104	0.086
igd	-0.296	-0.131	-0.369	-0.292	-0.272
let	0.044	0.023	0.042	0.076	0.112
rec	-0.061	-0.021	-0.106	-0.137	-0.096
cre	-0.217	-0.095	0.022	0.017	0.048
pro	-0.054	0.02	-0.049	0.012	-0.027

Table S5: **Principal component vector as combination of determinants**. For this analysis, we first rotated the feature matrix associated to a given comparator to the principal component coordinates. We then applied multiple step–wise logistic regression with each of the principal components as covariates. The model proposed is thus a combination of different principal components that we can express in the original (feature) coordinates. Results qualitatively agree with those of Tables 2–3, main text, and Tables S1–S4.

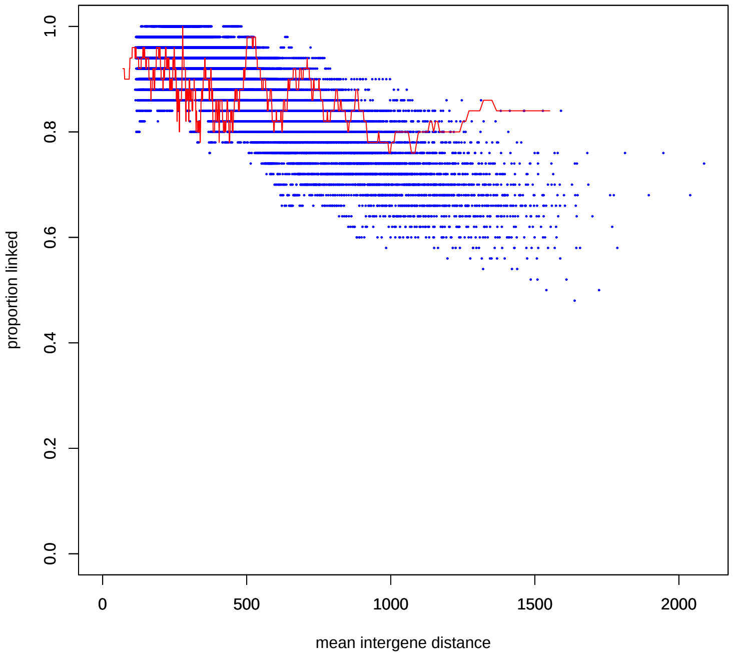

Figure S1: **Proportion of gene pairs conserved in** *S. castelli* **versus intergene distance in** *S. cerevisiae*. The profile of the rate of gene pairs conserved versus their current spacer in *S. cerevisiae* (red) or in simulants (blue) when comparing *S. cer* with *S. cas.* as comparator species. For the simulations the number of inversions to run was determined by comparing observed synteny conservation rates against inversion number as shown in figure 1, main text. We also restrict analysis to cases where both of the orthologues of the *S. cerevisiae* gene pair are on the same chromosome in the comparator species, as this fits better the simulant model and permits higher orthology certainty. Each data point in the real and simulant data represents the proportion of gene pairs from 50 showing conserved synteny, after the data is rank ordered by intergene distance.

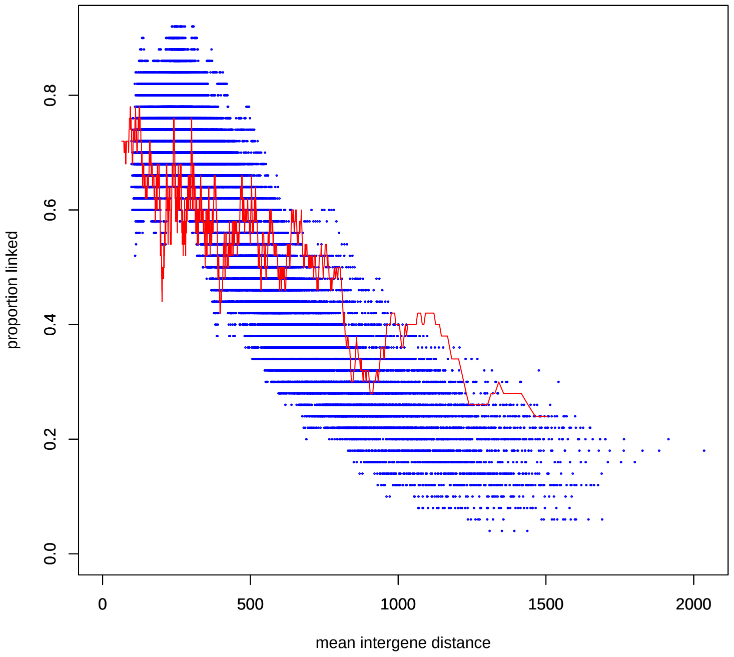

Figure S2: **Proportion of gene pairs conserved in** *K. waltii* **versus intergene distance in** *S. cerevisiae*. Data obtained as in Fig.S1.

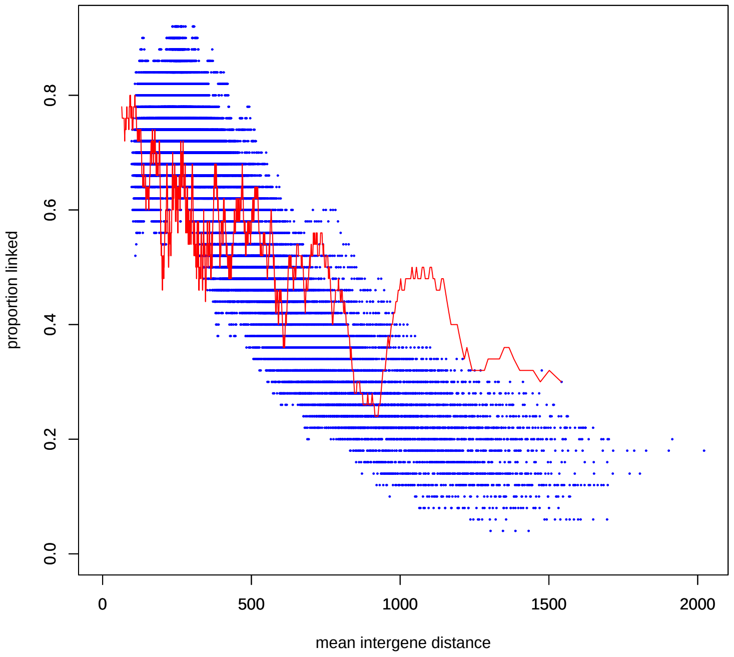

Figure S3: **Proportion of gene pairs conserved in** *K. lactis* **versus intergene distance in** *S. cerevisiae*. Data obtained as in Fig.S1.

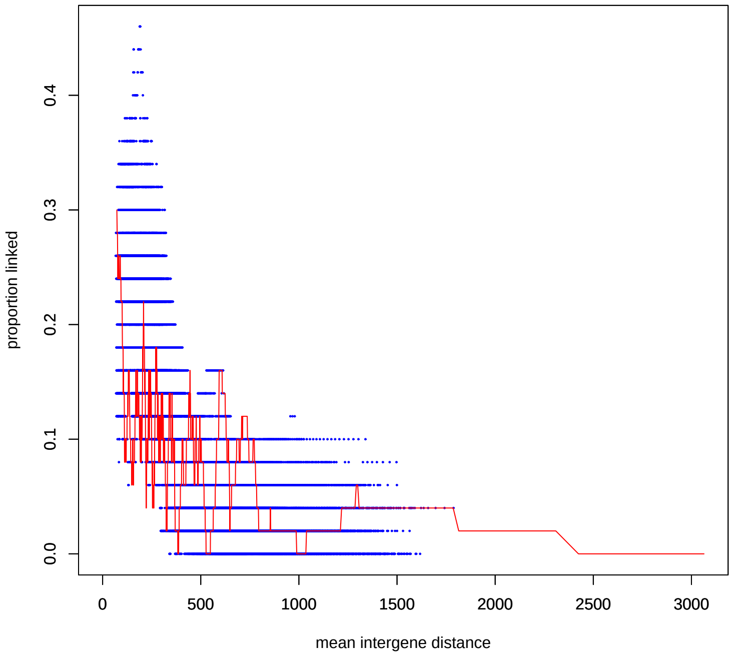

Figure S4: **Proportion of gene pairs conserved in *C. albicans* versus intergene distance in *S. cerevisiae*.** Data obtained as in Fig.S1.

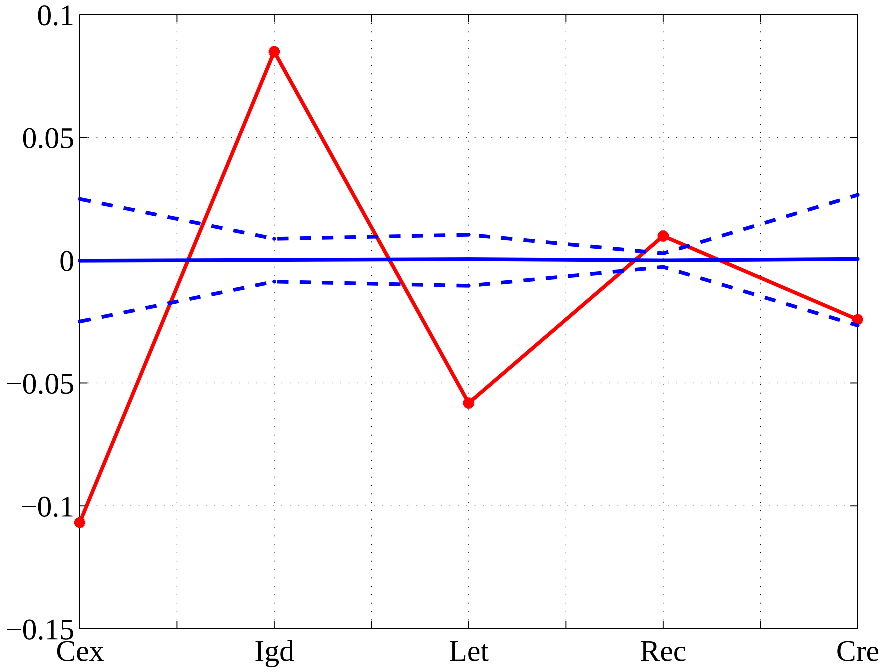

Figure S5: **Determinants of close non–adjacently conserved pairs vs. distant adjacently conserved pairs**. The difference between the ratio of determinant values of non–adjacently conserved genes in a close species to *S.cer.* (*S. cas.*) and those adjacently conserved in a distant species (*K. lac.*) is plotted in red for each predictor (line between points to help visualization). This ratio is defined as the quotient between the corresponding values of the close (distant) pairs and those of the adjacently conserved pairs in the close species, i.e., *S. cas*. We also plotted the null behaviour obtain by random sampling of the combined group, close and distant, preserving group size, 10000 times (mean, continuous blue line, $\pm$ 2 std, dashed blue lines). Behaviour was qualitatively robust for the cex, igd, and let with respect to Fig. 4, main text).
